# Genome Assembly and Pathway Analysis of Edible Mushroom *Agrocybe cylindracea*

**DOI:** 10.1016/j.gpb.2018.10.009

**Published:** 2020-06-17

**Authors:** Yuan Liang, Dengxue Lu, Sen Wang, Yuhui Zhao, Shenghan Gao, Rongbing Han, Jun Yu, Weili Zheng, Jianing Geng, Songnian Hu

**Affiliations:** 1 CAS Key Laboratory of Genome Sciences and Information, Beijing Institute of Genomics, Chinese Academy of Sciences, Beijing 100101, China; 2 Gansu Academy of Sciences, Lanzhou 730000, China; 3 University of Chinese Academy of Sciences, Beijing 100049, China

**Keywords:** Mushroom, Whole-genome sequencing, Mating type, Nutrients, Metabolic pathway

## Abstract

*Agrocybe cylindracea*, an edible **mushroom**, is widely cultivated for its abundance of **nutrients** and flavor, and many of its metabolites are reported to have beneficial roles, such as medicinal effects on tumors and chronical illnesses. However, the lack of genomic information has hindered further molecular studies on this fungus. Here, we present a genome assembly of *A. cylindracea* together with comparative genomics and pathway analyses of Agaricales species. The draft, generated from both next-generation sequencing (NGS) and single-molecule real-time (SMRT) sequencing platforms to overcome high genetic heterozygosity, is composed of a 56.5 Mb sequence and 15,384 predicted genes. This mushroom possesses a complex reproductive system, including tetrapolar heterothallic and secondary homothallic mechanisms, and harbors several hydrolases and peptidases for gradual and effective degradation of various carbon sources. Our pathway analysis reveals complex processes involved in the biosynthesis of polysaccharides and other active substances, including B vitamins, unsaturated fatty acids, and *N*-acetylglucosamine. RNA-seq data show that *A. cylindracea* stipes tend to synthesize carbohydrate for carbon sequestration and energy storage, whereas pilei are more active in carbon utilization and unsaturated fatty acid biosynthesis. These results reflect diverse functions of the two anatomical structures of the fruiting body. Our comprehensive genomic and transcriptomic data, as well as preliminary comparative analyses, provide insights into the molecular details of the medicinal effects in terms of active compounds and nutrient components.

## Introduction

*Agrocybe cylindracea*, belonging to the *Agrocybe* genus, is an edible mushroom that is very popular for the unique flavor and high nutritional content of its fruiting body. *A. cylindracea* is also regarded as a multipurpose food supplement due to the high levels of nutrients and bioactive compounds present in this species. Some studies have shown that active extracts of *A. cylindracea* have effects on various human diseases [1], [2].

Climatic and geographical diversity have resulted in the availability of a large variety of edible fungal species for domestication worldwide. As the largest mushroom clade, many Agaricales, such as *Agaricus bisporus*, *Laccaria bicolor* [3], and *Coprinopsis cinerea* [4], have been sequenced and analyzed at the whole-genome level. Sequencing analyses revealed that the genome sizes of Agaricales species range from 22.12 Mb [5] to more than 100 Mb, and the number of predicted genes is approximately 10–20 thousand. Many Agaricales species, including *Agrocybe aegerita* (*A. aegerita*), which belongs to the same genus as *A. cylindracea*, are rich in carbohydrate hydrolases and have the ability to degrade lignocellulosic biomass. Sequencing of *A. aegerita* also led to analyses of the carbohydrate-active genes and fruiting-related genes [6]. As a major cultivated fungal species, *A. cylindracea* is grown in many countries, including China, and has marked economic benefits for farmers. However, molecular research on *A. cylindracea* has been limited to lectins and active polysaccharide extracts.

Here, we present a genome assembly of *A. cylindracea* and a preliminary analysis of the complex reproductive system of this organism. Comparative analysis was conducted between the genome of *A. cylindracea* and the published genomes of 16 Agaricales fungi [3], [4], [6], [7], [8], [9], [10], [11], [12], [13], including edible mushrooms such as *Agaricus bisporus* and poisonous mushrooms such as *Galerina marginata*, to analyze the phylogeny of Agaricales species. We also attempted to construct a molecular framework of nutrient homeostasis, from element absorption to biosynthesis. Our work provides insights into the molecular details of *A. cylindracea* as both a food and potential medicine.

## Results

### Features of the *A. cylindracea* genome assembly

Based on the k-mer (17-mer) distribution, the size of our assembled *A. cylindracea* genome is estimated to be ~58.2 Mb (Figure S1). The two distinct peaks suggest high sequence heterozygosity, so this predicted genome size might be larger than the actual size. In addition, our genome characteristics estimation (GCE) result [14] indicated a hybridization rate of ~1.8%. To overcome the difficulty associated with the assembly of high-heterozygosity sequences, we combined data from both next-generation sequencing (NGS) and single-molecule real-time (SMRT) sequencing platforms and used the sequence assembler Platanus, which handles high-heterozygosity sequences better than the other options. A final genome assembly of 56.5 Mb was obtained, composed of 3790 scaffolds with a gap size of 0.9 Mb (1.7%). The scaffold size and contig N50 value are 547 kb and 48 kb, respectively (Table 1). Assembly validation shows a mapping rate of 92.5% based on the filtered paired-end reads. In addition, 86.7% of the assembled transcripts are aligned to the assembly (homology cutoff of 90%, Table S1). Our core eukaryotic genes mapping approach (CEGMA) analysis shows that 97.6% (242 of 248) of the core genes are present in the assembly, and 96.0% (238 of 248) are completely mapped.

**Table 1:** **Statistics of *A. cylindracea* genome assembly and gene prediction**

Our gene prediction yielded 15,384 predicted genes in the *A. cylindracea* genome assembly (Table 1), and among these genes, 68.43% (10,528), 90.17% (13,872), 55.36% (8516), 50.52% (7772), 2.85% (439), and 41.39% (6368) exhibit similarity to known annotated proteins in the NCBI-NR (identity > 0.3, Qcover > 0.5, Tcover > 0.5), InterPro, KEGG, Pfam, Carbohydrate-Active Enzyme (CAZy) (Table S2), and Gene Ontology (GO) databases.

Our sequence variation analysis yielded 871,977 variants (1.54% of the *A. cylindracea* genome), including 747,935 single-nucleotide variants (SNVs) (1.32% of the genome) and 124,042 indels and substitutions. Based on structural annotation of the genes, 269,636 SNVs in genes are predicted, and among these SNVs, 165,407 exonic SNVs are synonymous, whereas 104,229 are nonsynonymous, and others are outside the protein-coding regions (Table S3). In addition, we identified ~7.0 Mb (12.54% of the genome) of repeats, including interspersed repeats, simple repeats, and low-complexity repeats (Table S4). Excluding the unclassified repeats, the most abundant repeats are long interspersed nuclear elements (LINEs) (2.06%), followed by long terminal repeat (LTR) retroposons (1.28%).

### *A. cylindracea* possesses a complex mating system

**Figure 1:**
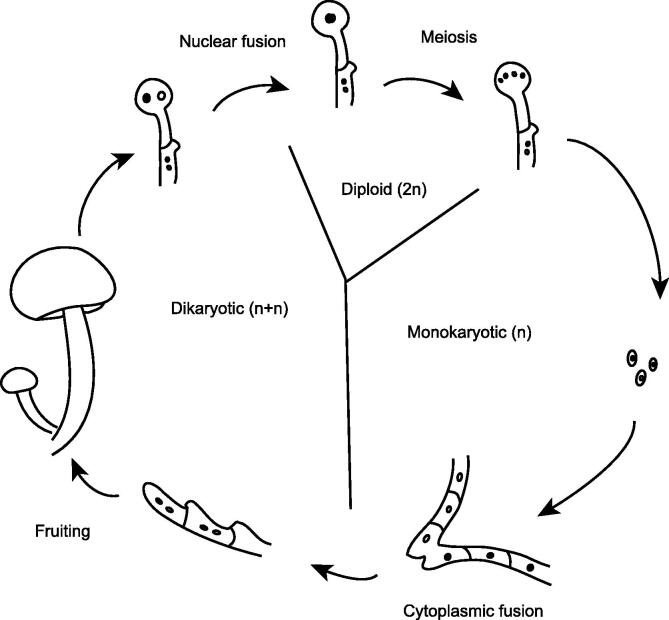
**Heterothallic sexual reproduction in basidiomycetes** Mature basidiospores germinate to form mycelia that eventually grow into fruiting bodies after cytoplasmic fusion. The fruiting bodies then produce a new generation of basidiospores via nuclear fusion and meiosis.

A single fungal species can have thousands of mating types, in contrast to the two sexes in animals or plants, which often leads to a large number of genetic polymorphisms for adaptation to ever-changing ecological environments. Fungi have evolved complex sexual processes, including homothallic (self-fertile) and heterothallic (self-sterile) mechanisms (Figure 1). A majority of basidiomycetes have a tetrapolar mating system of homogenic incompatibility [15], which is controlled by the two unlinked mating type (MAT) loci *A* and *B*. *A. cylindracea* has a single MAT *A* locus encoding two homeodomain transcription factors (HD1 and HD2), with a mitochondrial intermediate peptidase (MIP) nearby. This species has three pheromone-coding genes and five pheromone receptor-coding genes in the MAT *B* locus, which are clustered on contig394 (Table S5, Figure S2A). These pheromone receptors are characterized by a seven-transmembrane-domain structure [13], as identified with TMHMM (version 2.0) [16] (Figure S2B).

Many fungal species, such as *Saccharomyces cerevisiae* and *Chromocrea spinulosa*, exhibit MAT switching behavior, termed secondary homothallism, changing the original MAT and producing fertile offspring [15]. The identification of a switching protein, which plays a role in MAT switching [17], suggests the existence of such a process in *A. cylindracea*. It is also supported by the prediction of five silent information regulator (Sir)-coding sequences that participate in the silencing of the other MAT that is located near the expressed MAT locus (Table S5) [18]. Therefore, *A. cylindracea* is likely a typical tetrapolar basidiomycete with secondary homothallic behavior. This complex sexual reproduction system promotes gene exchange and survivability of *A. cylindracea*.

### Genome comparison between *A. cylindracea* and Agaricales

Based on predicted genes from Agaricales fungi (Table S6), 23,529 families of 274,314 proteins were clustered using OrthoMCL. In total, 8657 of 8939 families are shared with other fungi, and 282 families are unique (consisting of 949 genes) to *A. cylindracea*.

The phylogenetic tree was inferred based on RAxML from 315 orthologous proteins of the single-copy gene families (Figure S3). There is a close relationship between both the sequenced *Agrocybe* strains (*A. cylindracea* and *A. aegerita*), followed by *G. marginata*, *Hebeloma cylindrosporum* (*H. cylindrosporum*), and *Hypholoma sublateritium* (*H. sublateritium*). There are 732 orthologous blocks, containing 56.61% of the *A. cylindracea* genes (8709) and 60.36% of the *A. aegerita* genes (8520). The species-specific *A. cylindracea* genes, compared with the *A. aegerita* genes, are enriched in ion binding, protein binding, nitrogen compound metabolic process, and primary metabolic process (Figure S4A and B). Comparing all homologous genes among the *A. cylindracea* and four close relatives, we show that 53.68% (8258) of the *A. cylindracea* genes are homologues to the rest (Figure S5). The species-specific genes in *A. cylindracea* are significantly enriched in nitrogen compound metabolic process and binding functions (Figure S4C and D).

**Figure 2:**
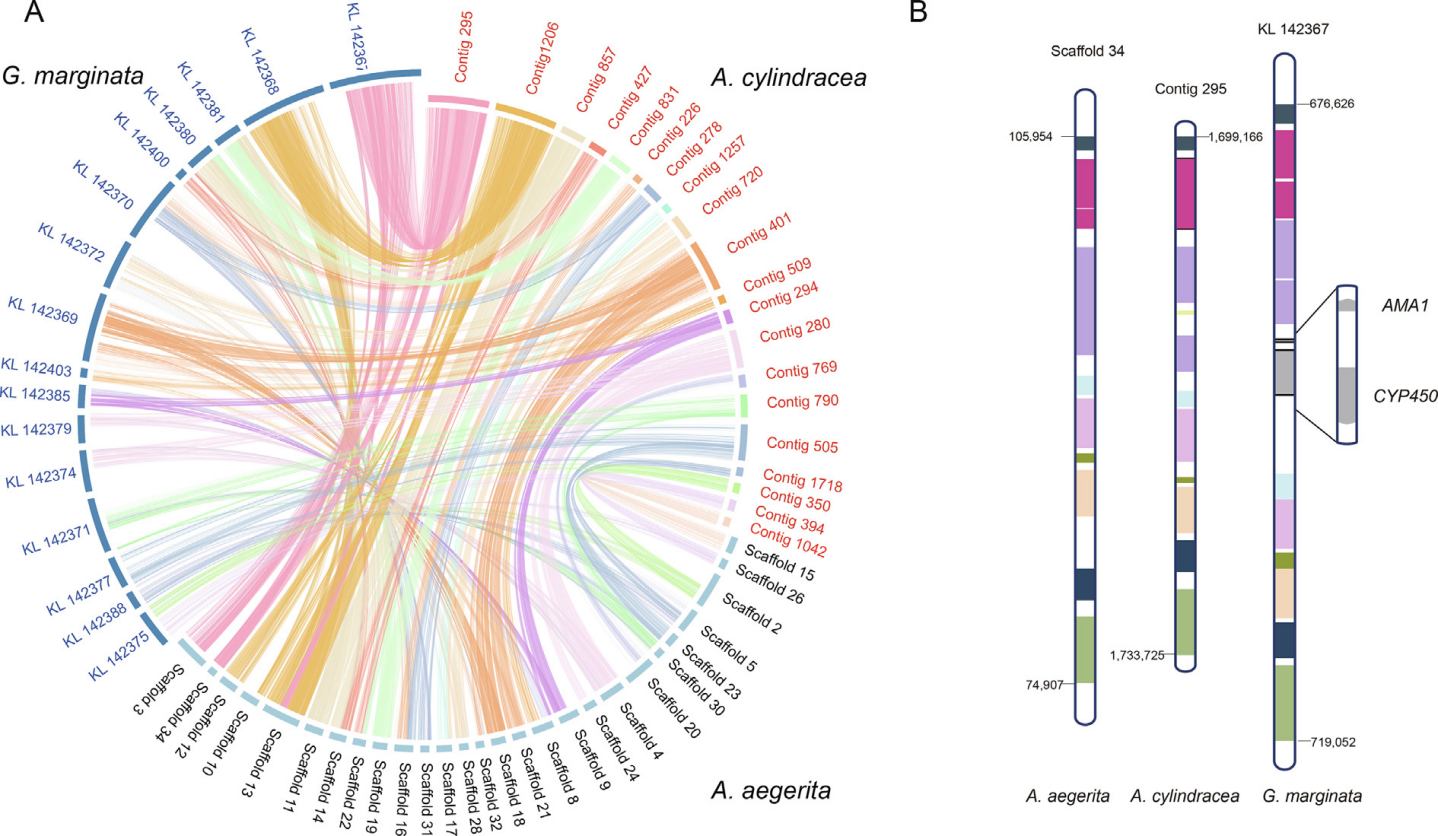
**Synteny analysis of *A. cylindracea*, *A. aegerita*, and *G. marginata*** **A.** High synteny among three species. *A. cylindracea* contigs are represented by colored blocks. *A. aegerita* and *G. marginata* are shown in light blue and dark blue, respectively. The homologous genes are connected with colored links that match the colors of the *A. cylindracea* contig blocks. **B.** High synteny among Contig 295 of *A. cylindracea*, Scaffold 34 of *A. aegerita*, and KL 142,367 of *G. marginata* (partial). Homologous genes are coded in the same color. The gray blocks represent genes that are absent in *A. cylindracea* and *A. aegerita*; these genes encode the toxin proteins α-amanitin (AMA1) and CYP450.

*A. cylindracea*, *A. aegerita*, and *G. marginata* are phylogenetically close, and *G. marginata* is an inedible mushroom. We compared the three genomes to identify the homologous regions (Figure 2A) and detected 825 orthologous blocks between *G. marginata* and *A. cylindracea* and 732 blocks between *A. aegerita* and *A. cylindracea* (Table S7). We found that the gene encoding the lethal protein α-amanitin (*AMA1*) in *G. marginata* is absent in *A. cylindracea* and *A. aegerita*. This gene is located on highly conserved blocks among the three species (Figure 2B). Mushrooms with the amanitin protein are highly toxic. Accidental ingestion by humans is known to lead to acute liver damage, kidney failure, and even death. Luo et al found that *G. marginata* does not contain any related toxin-coding sequences except two copies of the *AMA1* gene [19]. Whole-genome comparative analyses among the 17 mushroom genomes showed that no α-amanitin-coding gene could be detected in the genomes of edible mushrooms. The absence of amanitin in *A. cylindracea* and other edible mushrooms may be responsible for the edibility of these mushrooms.

### *A. cylindracea* utilizes exterior nutrition with unusual efficiency

Carbon and nitrogen are major energy resources for fungi. Cultivated *A. cylindracea* can obtain carbon from cultivation substrates, such as cottonseed hull, wheat straw, or sawdust. Based on the CAZy database annotation, 439 genes have been identified in *A. cylindracea* that are involved in carbon metabolic pathways, including 192 glycoside hydrolase (GH) superfamily members, 88 auxiliary activity (AA) enzymes, 30 carbohydrate esterases (CEs), 65 glycosyltransferases (GTs), 8 polysaccharide lyases (PLs), and 56 carbohydrate-binding module (CBM) superfamily members. Interestingly, 254 (58%) of these proteins are membrane-bound or secreted and possess a signal peptide domain (Figure S6), which suggests the ability of this organism to utilize external carbon sources.

*A. cylindracea* has genes that degrade cellulose. Cellobiohydrolases of the GH6 and GH7 families are correlated with the degradation of crystalline cellulose and are used to cleave cellulose to form disaccharide cellobiose [10], [20]. In our assembly, we identified 3 and 6 putative genes of the GH6 and GH7 families, respectively. GH1 (5 copies) and GH3 (11 copies), as beta-glucosidases, cleave cellobiose to form glucose and enhance the efficiency of cellulolytic enzymes [21]. Compared with other edible mushrooms, the GH1 family is expanded in *A. cylindracea* (Table S8). *A. cylindracea* also harbors the GH5 (17 copies), GH9 (1 copy), GH44 (1 copy), GH45 (1 copy), and lytic polysaccharide monooxygenase (LPMO) (17 copies) families, which are associated with cellulose deconstruction [10]. An additional 18 predicted genes containing the CBM1 domain (cellulose-binding module) were detected, which have been proposed to play important roles in cellulose degradation [20].

Similar to other white rot fungi, *A. cylindracea* degrades all polysaccharides of plant cell walls and lignin in addition to cellulose [22]. There are 5 putative peroxidases (AA2) in *A. cylindracea*, four of which are manganese peroxidases. The AA2 family includes class II lignin-modifying peroxidases such as lignin peroxidase (LiP), manganese peroxidase (MnP), and versatile peroxidase (VP), which are major enzymes for lignin degradation [23]. There are 26 CAZymes involved in the degradation of hemicellulose and pectin for carbon conversion (Table S9).

*A. cylindracea* degrades crude proteins from cultivation substrates as nitrogen source. Using the MEROPS database as a reference, we identified 2177 peptidases, including 256 proteins with a signal peptide domain. The most abundant peptidases are prolyl oligopeptidases (family S09/218 genes), from serine peptidases; Copia transposon peptidases (A11/133), from aspartic peptidases; and prolyl aminopeptidases (S33/130). The high abundance of peptidases suggests effective utilization of external nitrogen resources and high protein content.

In addition, *A. cylindracea* possesses a reliable transport system for absorbing nutrients, as supported by the fact that 359 nutrient transport-associated proteins have been identified based on Pfam annotation, including ABC and MFS family transporters that are responsible for transporting oligopeptides, amino acid sugars, and metal ions.

### Nutrient abundance of *A. cylindracea* and reconstruction of the metabolic processing networks of this species

**Figure 3:**
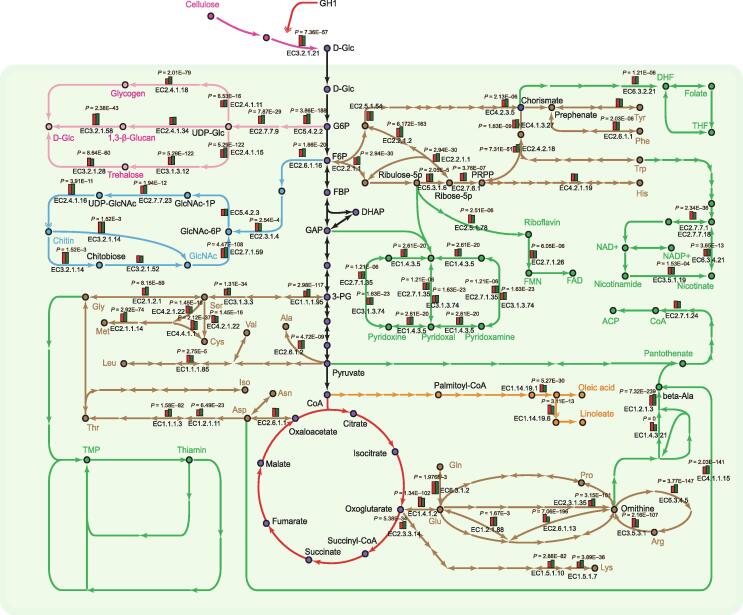
**Nutrient biosynthetic pathways of *A. cylindracea*** The arrows represent the enzymes that were identified in *A. cylindracea,* and each dot represents the metabolite that was generated by enzymatic catalysis. Different colors represent different metabolic pathways of nutrient substances, including carbohydrate (pink), GlcNAc (blue), B vitamins (green), amino acids (brown), and unsaturated fatty acids (yellow). Highly expressed enzyme-coding genes in fruiting bodies are represented as column diagrams (red columns on the left represent the gene expression level in pilei, green columns on the right represent the gene expression level in stipes). Enzyme EC numbers are provided below the respective column diagrams and *P* values of DEGs are provided above the respective column diagrams. The smaller the *P* value is, the more significant the difference in gene expression between the pilei and stipes of the *A. cylindracea* fruiting bodies. Double-headed arrows represent the expanded genes. DEG, differentially expressed gene; Glc, glucose; G6P, glucose 6-phosphate; F6P, fructose 6-phosphate; FBP, fructose-1,6-bisphosphatase; GAP, glyceraldehyde-3-phosphat; DHAP, dihydroxyacetone phosphate; 3-PG, 3-phospho-D-glycerate; CoA, coenzyme A; GlcNAc-1P, N-acetylglucosamine-1-phosphate; DHF, dihydrofolate; THF, tetrahydrofolate; NAD, nicotinamide adenine dinucleotide; NADP, nicotinamide adenine dinucleotide phosphate; FMN, flavin mononucleotide; FAD, flavin adenine dinucleotide; ACP, acyl carrier protein; TMP, thiamin monophosphate.

To elucidate the molecular mechanism of nutrient metabolism, we constructed the metabolic processing networks, including the carbon cycle and biosynthetic pathways of amino acids and other nutrients (Figure 3). Almost all the genes involved in the TCA cycle and glycolysis pathway are highly expressed (top 10%) in fruiting bodies, revealing that *A. cylindracea* can utilize carbon sources efficiently to provide energy and materials for the biosynthesis of other nutrients, such as amino acids, B vitamins, polysaccharides, and unsaturated fatty acids.

Beta-glucans, especially beta-1,3-glucans, exhibit high biological activity in immunomodulatory, anti-inflammatory, and antitumor processes [24] and are abundant in *A. cylindracea*, in which all the 1,3-β-glucan synthesis-associated enzymes were identified, together with ten predicted beta-1,6-glucan biosynthesis-associated proteins, KRE6 and SKN1 (Table S10) [25]; However, the detailed mechanism underlying beta-1,6-glucan synthesis remains hypothetical. Compared with other edible mushrooms, beta-1,6-glucan synthesis-associated proteins are expanded in *A. cylindracea*. In the chitin synthesis pathway, chitin synthases (EC3.2.1.14) are both highly expressed and expanded. Moreover, chitinase, which hydrolyzes chitin to produce *N*-acetylglucosamine (GlcNAc), is expressed at high levels in the *A. cylindracea* fruiting bodies. These expanded and highly expressed genes indicate the ability of *A. cylindracea* to produce functionally active carbohydrate.

*A. cylindracea* is also an ideal source of amino acids, unsaturated fatty acids, and B vitamins. The nutrients in *A. cylindracea* are diverse and highly active (Figure 3). Highly expressed enzymes, such as glutamine synthetase and aspartate aminotransferase, may enhance the umami taste of *A. cylindracea* fruiting bodies [26]. We reconstructed the synthetic pathway of six B vitamins and identified several biotin synthesis-associated enzymes, including 8-amino-7-oxononanoate (AON) synthase (EC2.3.1.47), DAN synthase (EC2.6.1.62), desthiobiotin (DTB) synthase (EC6.3.3.3), and biotin synthase (EC2.8.1.6). In addition, delta-12-desaturase is expanded in *A. cylindracea*, indicating increased levels of the essential polyunsaturated fatty acid linoleic acid [27].

### The secondary metabolism in *A. cylindracea*

Edible mushrooms are known for their abundance of secondary metabolites [28]. We identified 232 categories of enzymes involved in “biosynthesis of secondary metabolites” (ko01110) by KEGG pathway mapping (Table S11). We also analyzed secondary metabolite gene clusters by using the antiSMASH web-based analysis platform (version 4.1.0) [29]. There are 15 gene clusters encoding key enzymes in terpene biosynthesis (Table S12). The terpene synthase cluster is the largest cluster in *A. cylindracea*. In fungi, terpenoids are derived from dimethylallyl diphosphate (DMAPP) and isopentenyl diphosphate (IPP) [30]. We identified 15 categories of enzymes involved in “terpenoid backbone biosynthesis” (Table S13); the enzymes produce DMAPP and IPP from acetyl-CoA via the mevalonate pathway. There are 7 categories of enzymes in the “ubiquinone and other terpenoid-quinone biosynthesis” pathway, indicating the ability of *A. cylindracea* to synthesize ubiquinone (Table S13).

In addition, 15 enzymes were identified as being involved in steroid biosynthesis according to the KEGG database (Table S13). In particular, we identified a single-copy gene that encodes lanosterol synthase (LSS), which synthesizes lanosterol, a common cyclic intermediate of triterpenoids and ergosterol (provitamin D2) [31]. *A. cylindracea* also has 3 type I polyketide (T1pk) clusters containing 24 related genes. Polyketide synthases (PKSs) are known to be responsible for both aromatic and highly reduced polyketide metabolites [32]. We also identified 2 gene clusters with 15 putative genes associated with indole biosynthesis in *A. cylindracea*. A total of 28 secondary metabolite gene clusters were detected (Table S12). This finding indicated the potential ability of *A. cylindracea* to synthesize secondary metabolites with biological activities.

### Gene expression analysis indicates different biological functions between pilei and stipes in *A. cylindracea*

**Figure 4:**
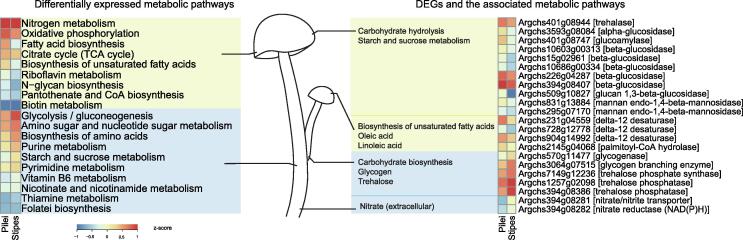
**Differential expression in metabolic pathways and DEGs between pilei and stipes of *A. cylindracea*** The columns represent pilei and stipes, and the rows represent metabolic pathway groups (left) or DEGs (right) based on KEGG annotation. The pathways and genes with higher expression levels in pilei than in stipes are marked with a yellow background, while those with higher expression levels in stipes than in pilei are marked in blue.

Our transcriptomic study yielded 2897 differentially expressed genes (DEGs) in the fruiting bodies (*P* < 0.05, |log_2_(fold-change)| > 1), including 1744 upregulated genes in pilei and 1153 in stipes (Figure 4). Overall, the upregulated pathways in pilei were mostly metabolic pathways, including *N*-glycan biosynthesis, oxidative phosphorylation, and biosynthesis of unsaturated fatty acids. The highly expressed enzyme mannan endo-1,4-beta-mannosidase can hydrolyze 1,4-beta-D-mannosidic linkages in mannans to form mannose, which is a unit of *N*-glycan. Key enzymes involved in unsaturated fatty acid (oleic acid and linoleic acid) synthesis, namely, delta-9-desaturase (EC1.14.19.1) and delta-12-desaturase (EC1.14.19.-), are also highly expressed in pilei. Interestingly, many annotated carbohydrate hydrolases, including beta-1,3-glucan, maltose, glycogen, trehalose, and dextrin hydrolases, are significantly upregulated in pilei.

*A. cylindracea* stipes show increased expression levels of several carbohydrate and amino acid metabolic pathways. In contrast to the genes in pilei, the upregulated genes in stipes are mostly involved in carbohydrate biosynthesis, such as those involved in the biosynthesis of dextrin, maltose, glycogen, and trehalose. A key enzyme involved in glycogen synthesis, namely, glycogen-branching enzyme (EC2.4.1.18) [33], as well as trehalose phosphate synthase (EC2.4.1.15) and trehalose phosphatase (EC3.1.3.12), which are involved in disaccharide trehalose synthesis, are all upregulated in stipes. Furthermore, genes encoding nitrate transporters and nitrate reductases in nitrogen metabolic pathways are also upregulated in stipes, indicating the existence of additional pathways in stipes for nitrogen source utilization.

## Discussion

The *A. cylindracea* genome is highly heterozygous, most likely due to outcrossing during cultivation and the complex reproductive system of this organism. During heterothallic sexual reproduction (Figure 1), the dikaryotic cell stage is maintained for a long duration after cell fusion instead of processing via nuclear fusion immediately. To regulate nuclear fusion, *A. cylindracea* could carry out a tetrapolar mating mechanism of homogenic incompatibility that is controlled by two unlinked loci. The two MAT loci are both multiallelic [15], promoting outbreeding and accumulation of genetic polymorphisms. Meanwhile, homokaryotic strains may produce fertile progenies via MAT switching. This switching model indicates the high reproductive capacity of *A. cylindracea*, and homothallism likely ensures the transfer of genetic information to the next generation. Understanding the complexity and diversity of fungal sexual reproduction systems is essential for the study of the biodiversity and genetic breeding of *A. cylindracea*.

The *A. cylindracea* genome assembly shows high-level synteny with the poisonous mushroom *G. marginata*, which is supported by sequence conservation. Nevertheless, the absence of the α-amanitin-coding gene may be a cause of the nontoxicity of this species, in contrast to *G. marginata,* which has two copies of this gene [19]. In addition, species*-*specific *A. cylindracea* genes, compared with genes in the most closely related species, are enriched in nitrogen compound metabolic processes and protein binding functions, and the GH1 carbohydrate hydrolase family is clearly expanded in *A. cylindracea* compared to other edible mushrooms. These genomic changes suggest the increased nitrogen and carbon source utilization capacity of *A. cylindracea*.

*A. cylindracea* absorbs and processes external nutrients to form small molecules by producing a large number of hydrolases and proteases. We reconstructed the biosynthetic pathways of 20 amino acids, which assure a high protein content and rich flavor of *A. cylindracea* fruiting bodies. *A. cylindracea* possesses a large number of enzymes that synthesize B vitamins, including folic acid, which is good for pregnant women. Moreover, *A. cylindracea* can synthesize various polysaccharides with antitumor activity, which might be further applied in clinical treatment. In addition, GlcNAc, usually extracted from crab and shrimp shells, is popularly used as an auxiliary natural drug with low side effects for arthritis patients. However, for patients who are allergic to seafood, *A. cylindracea* may be a better alternative. *A. cylindracea* has some unsaturated fatty acids that contribute high blood cholesterol levels. In particular, the linoleic acid synthesis-associated enzyme exhibits both expansion and high expression levels, which most likely indicates high levels of linoleic acid in *A. cylindracea* [27]. Pathway analyses show different functional characteristics in different parts of the *A. cylindracea* fruiting body. Nutrient biosynthesis pathways, such as unsaturated fatty acid and N-glycan biosynthetic pathways, are highly active in pilei. Pilei likely acquire carbon and energy by degrading carbohydrate. Conversely, stipes exhibit increased expression of disaccharide and polysaccharide biosynthesis pathways for carbon fixation. Active amino acid metabolism and extracellular nitrate absorption in stipes aids the accumulation of nitrogen sources. *A. cylindracea* stipe tissues prefer to store energy, while the pilei likely serve as nutrient factories.

In this study, we describe an assembly of the *A. cylindracea* genome and construct a detailed pathway for the synthesis of nutrients and flavor-related substances (some amino acids and saccharides). The highly expressed glutamine synthetase in *A. cylindracea* is a central enzyme in nitrogen assimilation and biosynthesis of glutamine [34]. Overexpression of delta-12-desaturase, which is expanded in *A. cylindracea,* can increase the linoleic acid content [35]. These enzymes may be key enzymes for industrial synthesis of natural products and for increasing the levels of active substances. The detailed metabolic analyses provide a theoretical basis for further research. Of course, the conclusion should be validated by *in vitro* and *in vivo* experiments. Genome sequences and transcriptomic analyses make breeding for high-quality strains and improved yields increasingly realistic and provide information regarding the sequences and expression characteristics of the key genes for research on biological synthesis.

## Conclusion

The *A. cylindracea* genome assembly and transcriptomic analysis provide further information for basic research on cultivation and drug development. A further refined *A. cylindracea* reference genome will be an important genomic resource for studies on sexual reproduction systems, genetic diversity, and domestication trait selection. Genomic analyses will allow researchers to identify the related genes that affect the quality, nutrient content, and biological efficiency of *A. cylindracea*. With more sequencing of different strains, DNA barcoding could be exploited for screening the excellent or specific strains rapidly and stably.

## Materials and methods

### DNA library construction and sequencing

We chose the *A. cylindracea* AC9 strain for sequencing and analysis. The AC9 strain was derived from wild strains picked by Dengxue Lu in Chaoyang village, Xingcun town, Wuyishan city (E 117°49′45″, N 27°38′26″) in 1999 and cultivated by the Gansu Provincial Academy of Sciences Institute of Biology in China. The wild strains grew on the rotten roots of *Camellia oleifera* trees. We isolated the strains at the top of the stipes by tissue isolation. After years of breeding and selection, the biological efficiency reached 75%, with good taste and appearance.

The genomic DNA libraries of *A. cylindracea* mycelia with different insert sizes were constructed and sequenced with the Illumina HiSeq 2000 and PacBio RS II platforms in Beijing Institute of Genomics, including three paired-end libraries (insert sizes: 300 bp, 500 bp, and 500 bp), two mate-pair libraries (8 kb and 10 kb), and one PacBio library (Table S14).

### Extraction of high-quality reads

We filtered adaptors, low-quality bases (cutoff score ≤ Q20), and PCR duplications of the raw data using in-house Perl scripts [36]. Contaminating reads were subsequently removed by searching bacterial databases from NCBI. A total of ~121× paired-end reads and ~27× mate-pair reads were obtained for assembly and analysis (Table S14).

### Genome size estimation

Jellyfish (version 2.1.3) [37] was used to count the 17-mer frequency of paired-end DNA libraries (one 300-bp and two 500-bp libraries). Based on the frequency, two formulas were applied to estimate the genome size of *A. cylindracea*: M = N × (L − K + 1)/L and G = T/N (where M represents the peak of distribution, N represents the actual sequencing depth, L represents the average read length, K represents the k-mer length, and G represents the estimated genome size) [38].

### Genome assembly and evaluation

High-quality paired-end reads were assembled into contigs by the *de novo* assembler Platanus [39], which is designed for high-heterozygosity genomes. We used Platanus on paired-end and mate-pair reads to construct scaffolds from contigs and fill gaps. The remaining gaps were filled with Gapfiller [40] and PBjelly [41] using PacBio long reads. To remove any potential repeat sequences derived from the heterozygosity, we aligned the scaffolds to themselves using BLAST searching (coverage ≥ 90%, identity ≥ 95%, alignment length ≥ 90%), and duplicated sequences were removed. To identify possible bacterial contamination, the assembled scaffolds were aligned to the bacterial database from NCBI, removing the 100% matched sequences. Scaffolds with sequences ≥ 500 bp were used for subsequent analyses.

To evaluate the genome assembly, three approaches were used in our study: (1) raw data mapping, where we calculated mapping rates by mapping paired-end reads back to the assembly; (2) core gene mapping, where we used CEGMA [42] with default parameters to evaluate 248 conserved core eukaryotic genes for completeness by aligning them to the assembly; and (3) transcript mapping, where we mapped *de novo*-assembled transcripts to the assembly.

### Detection of heterozygous variations

The high-quality reads were mapped to the assembly by BWA (version 0.7.15-r1140), and duplicate reads were removed using MarkDuplicates from PICARD tools (version 1.119) (http://picard.sourceforge.net). Heterozygous variations (SNVs and indels) were detected with SAMtools (version 1.3.1) and GATK (version 3.5) [43]. SNVs with high coverage (DP ≥ 10) were considered reliable and annotated with ANNOVAR [44].

### Repetitive sequence annotation

RepeatModeler (http://www.repeatmasker.org/RepeatModeler.html, version 1.0.7), which employs two programs, namely, RECON and RepeatScout, was applied with default settings to construct a *de novo* repetitive library from the assembly, which was further annotated.

### Gene prediction and annotation

Three strategies were used for gene prediction: (1) *Ab initio* gene prediction, where gene models were predicted *ab initio* based on Augustus (version 3.0.1) [45], GlimmerM (version 3.0.2) [46], and SNAP (version 2013-11-29) [47] with a transcriptome-based training set constructed using autoAug.pl and PASA (version 2.0.0) [48]; (2) homology-based prediction, where fungal protein sequences from SwissProt were used as evidence for homology-based prediction by SPLAN (version 2.0.6) [49]; and (3) transcriptome-based prediction, where transcripts were aligned to the assembly, and exons and introns were determined. Finally, the gene structures from the three strategies were combined to generate a reliable gene set using EvidenceModeler (EVM, version 1.1.1) [50].

Predicted genes were annotated by a BLAST search against NCBI databases with an E-value cutoff of 10^−5^. InterProScan [51] was used to predict motifs and domains, as well as GO terms. Metabolic pathways were reconstructed using the KEGG database (http://www.kegg.jp/kegg/). Carbohydrate-active and peptidase enzymes were classified by aligning predicted protein sequences against the CAZy and MEROPS databases with an *E*-value cutoff of 10^−5^. Pheromone precursors were predicted by TransDecoder (https://transdecoder.github.io/) and the Pfam database in the flanking regions of pheromone receptors.

### RNA-seq analysis

cDNA libraries of *A. cylindracea* AC9 mycelia, constructed from pileus and stipe RNAs, were sequenced on the Illumina HiSeq 2000 platform (Table S15) in Beijing Institute of Genomics. Adaptor sequences and low-quality reads (cutoff score ≤ Q20) were trimmed with in-house Perl scripts. The reads from *A. cylindracea* AC9 mycelia were *de novo* assembled to build transcripts with Trinity [52] (Table S16).

The preprocessed RNA-seq reads from stipes and pilei were mapped to the *A. cylindracea* genome assembly. Due to the high heterozygosity of the genome, Novoalign (http://www.novocraft.com/) was used to obtain improved alignment results. FPKM was calculated using Cufflinks (version 2.0.2) [53]. When FPKM values were sorted from large to small, the top 10% of the genes were considered to be highly expressed genes. Read counts were calculated based on the HTseq count, and DEGs between stipes and pilei were identified with DEseq (*P* < 0.05, |fold change| > 2). Triple duplication was used to reduce sampling bias (Figure S7A–F).

### Gene family construction and phylogeny reconstruction

The protein sequences of *A. cylindracea*, 16 Agaricales fungi and *Serpula lacrymans* [54] (Table S6) with lengths ≥ 30 aa were used to calculate pairwise similarities by all-to-all BLAST (E-value ≤ 10^−5^). Gene families were constructed using the OrthoMCL (v2.0.9) [55] pipeline with an inflation value of 2.0.

Single-copy gene families were extracted for phylogenetic analysis. Protein alignments of each family were performed with MUSCLE [56] and concatenated with in-house Perl scripts. The best evolution models were selected based on concatenated alignments without gaps for amino acids with ProtTest (version 3.4) [57]. The maximum-likelihood tree was inferred using RAxML (version 8.0.24) [58] with the model LG + I + G + F (200 bootstrapping replicates) and *Serpula lacrymans* as the outgroup. Orthologous gene blocks between *A. cylindracea* and other fungi were identified using MCscanX [59] with default settings.

### Metabolic pathway reconstruction

We mapped annotated *A. cylindracea* enzymes onto metabolic pathways using the “basic pathway mapping tool” from the KEGG website. We selected the most important nutrient metabolic pathways, including the carbon cycle and B vitamin and polysaccharide metabolic pathways, for further analysis. In addition, we checked the *A. cylindracea*-specific pathways via manual inspection and constructed a network of energy and nutrient pathways.

## Strain availability

The strain has been submitted to the Agricultural Culture Collection of China (ID: ACCC 53291), which is accessible at http://www.accc.org.cn/, and the China General Microbiological Culture Collection Center (ID: CGMCC5.2198), which is accessible at http://www.cgmcc.net/.

## Data availability

The raw sequence data reported in this paper have been deposited in the Genome Sequence Archive [60] at the National Genomics Data Center, Beijing Institute of Genomics, Chinese Academy of Sciences / China National Center for Bioinformation (GSA: CRA001724), and are publicly accessible at http://bigd.big.ac.cn/gsa. The data analyzed during this study are deposited at NCBI under BioProject PRJNA353318 (GenBank: MPNV00000000). Phylogenetic data have been deposited in the TreeBASE repository (ID: 22964), which is accessible at http://www.treebase.org/.

## CRediT author statement

**Yuan Liang:** Formal analysis, Visualization, Writing-original draft, Writing-review & editing. **Dengxue Lu:** Resources, Conceptualization, Writing-review & editing. **Sen Wang:** Formal analysis, Visualization, Writing-original draft. **Yuhui Zhao:** Resources. **Shenghan Gao:** Formal analysis. **Rongbin Han:** Resources. **Jun Yu:** Writing-review & editing. **Weili Zheng:** Resources, Supervision. **Jianing Geng:** Supervision, Writing-review & editing. **Songnian Hu:** Conceptualization, Supervision. All the authors read and approved the final manuscript.

## Competing interests

The authors declare that they have no competing interests.

## Supplementary material

Supplementary Figure S1 Frequency distribution of 17-mers The two peak depths in *A. cylindracea* are ~51 and ~101. The first peak is a hybrid peak, which indicates high heterozygosity of *A. cylindracea*. Based on the second peak, we estimated that the genome size of *A. cylindracea* is 58.2 Mb.

Supplementary Figure S2 Mating type loci of *A. cylindracea***A.** A single MAT *A* locus of genes encoding HD1 (pink box) and HD2 (green) homeodomain proteins and a MAT *B* locus of genes encoding pheromones (gray) and pheromone receptors (yellow) are positioned on different scaffolds. The blue box represents the gene encoding mitochondrial intermediate peptidase (MIP). **B.** Seven-transmembrane feature of pheromone receptors.

Supplementary Figure S3 Phylogenetic analysis of *A. cylindracea* and 16 Agaricales fungi based on single-copy orthologous genes. The phylogenetic tree was inferred based on RAxML from 315 orthologous proteins of the single-copy gene families. *Serpula lacrymans* serves as an outgroup. There is a close relationship between *A. cylindracea* and *A. aegerita*.

Supplementary Figure S4 GO enrichment of *A. cylindracea*-specific genes compared with *A. aegerita*, *G. marginata*, *H. cylindrosporum*, and *H. sublateritium* Each circle represents a family of GO terms, and the circle size reflects the number of enrichment genes. The arrows point from parental GO terms to the filial GO terms. The darker the color is, the more significant the enrichment is. **A.** GO enrichment analysis of *A. cylindracea*-specific genes compared with *A. aegerita* in the BP category. **B.** GO enrichment analysis of *A. cylindracea*-specific genes compared with *A. aegerita* in MF category. **C.** GO enrichment analysis of *A. cylindracea*-specific genes compared with the other four fungi in the BP category. **D.** GO enrichment analysis of *A. cylindracea*-specific genes compared with the other four fungi in the MF category. BP, biological process; MF, molecular function.

Supplementary Figure S5 Homologous genes shared among *A. cylindracea*, *A. aegerita*, *G. marginata*, *H. cylindrosporum*, and *H. sublateritium* The triangle represents the total genes of *A. cylindracea*. The ovals represent the shared homologous genes of *A. aegerita* (red), G. marginata (green), H. cylindrosporum (blue), and H. sublateritium (yellow) with *A. cylindracea*.

Supplementary Figure S6 Signal peptide ratio in *A. cylindracea* The vertical axis represents the percentage of proteins with signal peptides, and the horizontal axis represents functional proteins annotated by different databases: “All” represents all the predicted proteins in *A. cylindracea*; “CAZy” represents the carbohydrate hydrolytic enzymes in *A. cylindracea* based on the CAZy database; “MEROPS” represents the proteases in *A. cylindracea* based on the MEROPS database; “Transporter” represents the transporter-related proteins according to gene annotation in NCBI-NR database. The numbers above the columns represent the number of proteins with signal peptides in various databases.

Supplementary Figure S7 Consistency between biological replicates of the *A. cylindracea* transcriptomic data. Both pilei (**A, B, and** C) and stipes (**D, E, and F**) of *A. cylindracea* fruiting bodies have three biological replicates. Graphics reveal a very high consistency between biological replicates: pilei_1, pilei_2, and pilei_3 are the biological replicates of the pilei, and stipes_1, stipes_2, and stipes_3 represent the biological replicates of the stipes. FPKM represents the gene expression level. The most highly expressed gene in pilei encodes aegerolysin (Aa-Pri1), while the most highly expressed gene in stipes encodes calmodulin.
