# Supplementary figures and images for: Genome Assembly and Pathway Analysis of Edible Mushroom Agrocybe cylindracea
Source: Genomics Proteomics Bioinformatics. 2020 Jun 17;18(3):341–51. doi: 10.1016/j.gpb.2018.10.009 (PMC7801210; doi:10.1016/j.gpb.2018.10.009)

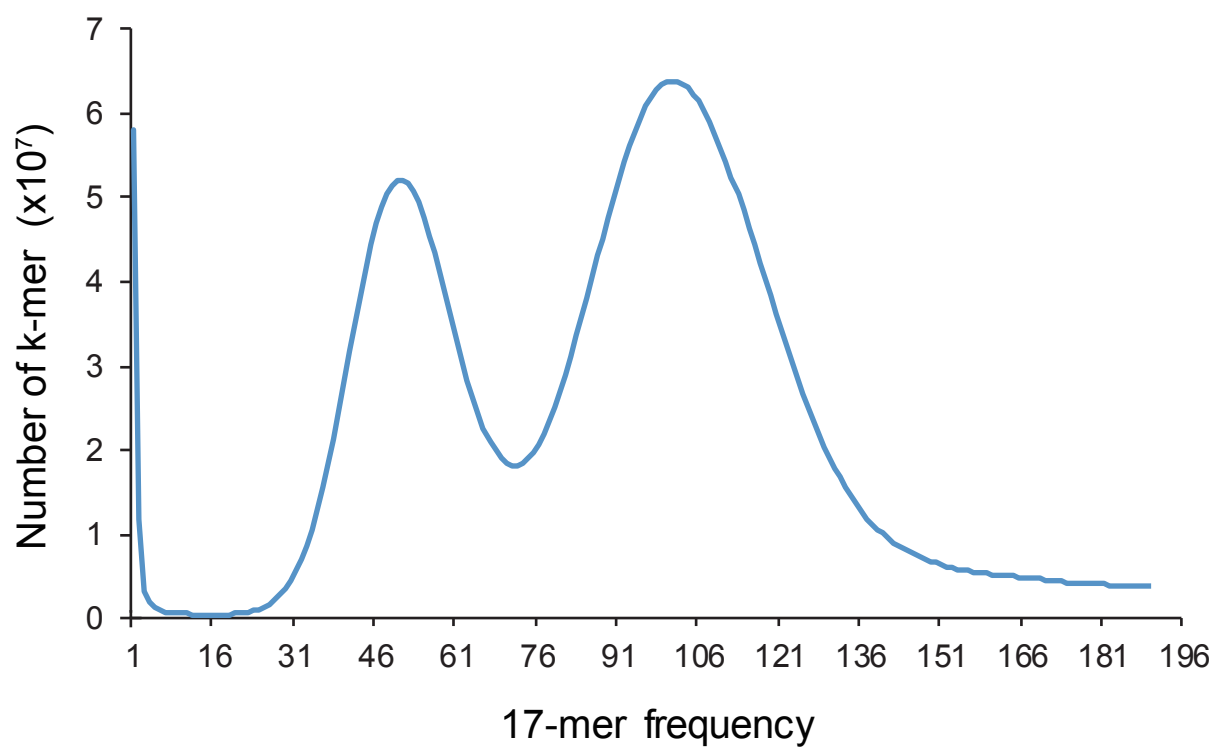

Supplement: Supplementary Figure S1 — Frequency distribution of 17-mers The two peak depths in A. cylindracea are ~51 and ~101. The first peak is a hybrid peak, which indicates high heterozygosity of A. cylindracea. Based on the second peak, we estimated that the genome size of A. cylindracea is 58.2 Mb. [file mmc1.pdf]

A

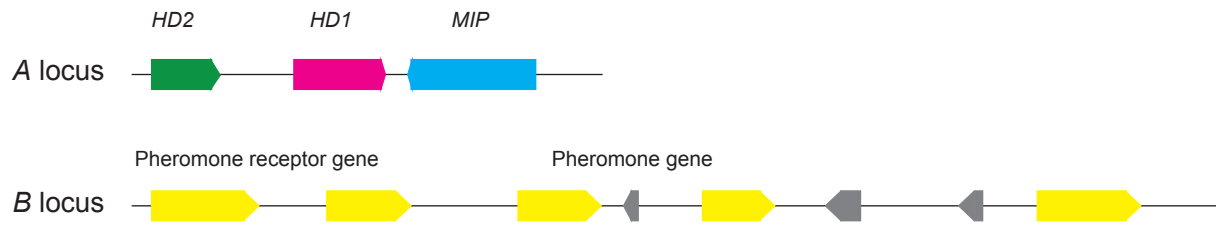

B

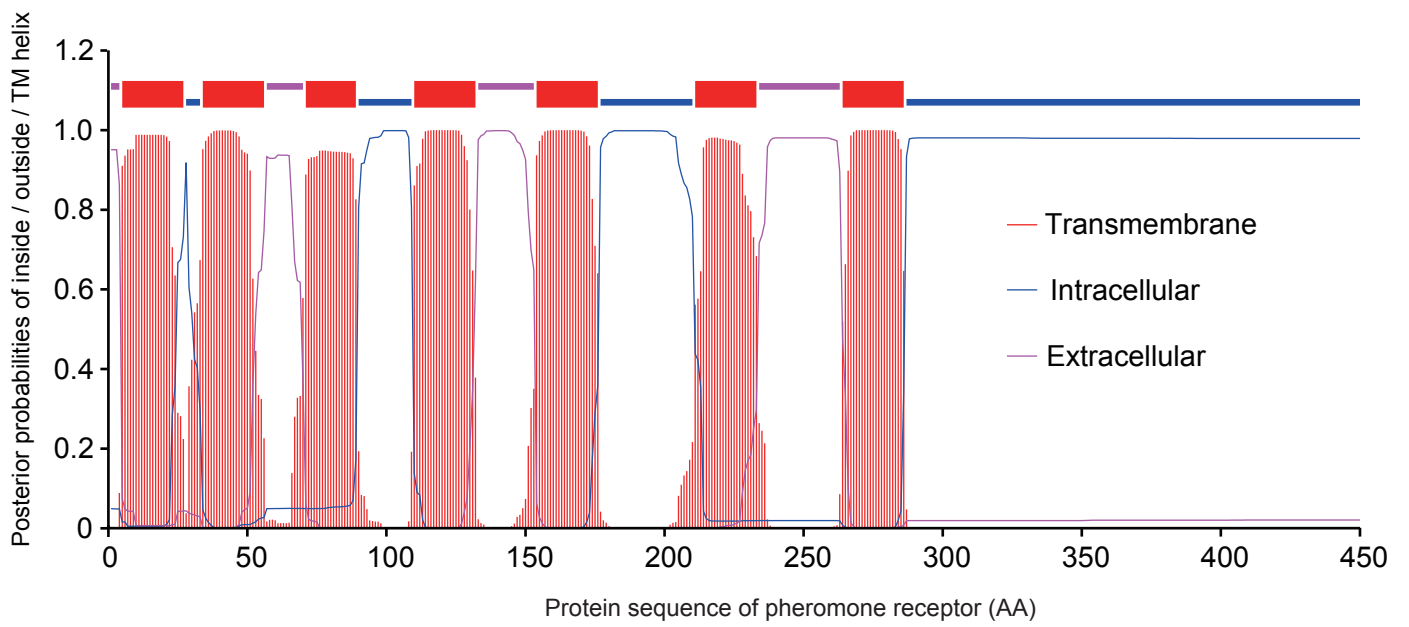

Supplement: Supplementary Figure S2 — Mating type loci of A. cylindraceaA. A single MAT A locus of genes encoding HD1 (pink box) and HD2 (green) homeodomain proteins and a MAT B locus of genes encoding pheromones (gray) and pheromone receptors (yellow) are positioned on different scaffolds. The blue box represents the gene encoding mitochondrial intermediate peptidase (MIP). B. Seven-transmembrane feature of pheromone receptors. [file mmc2.pdf]

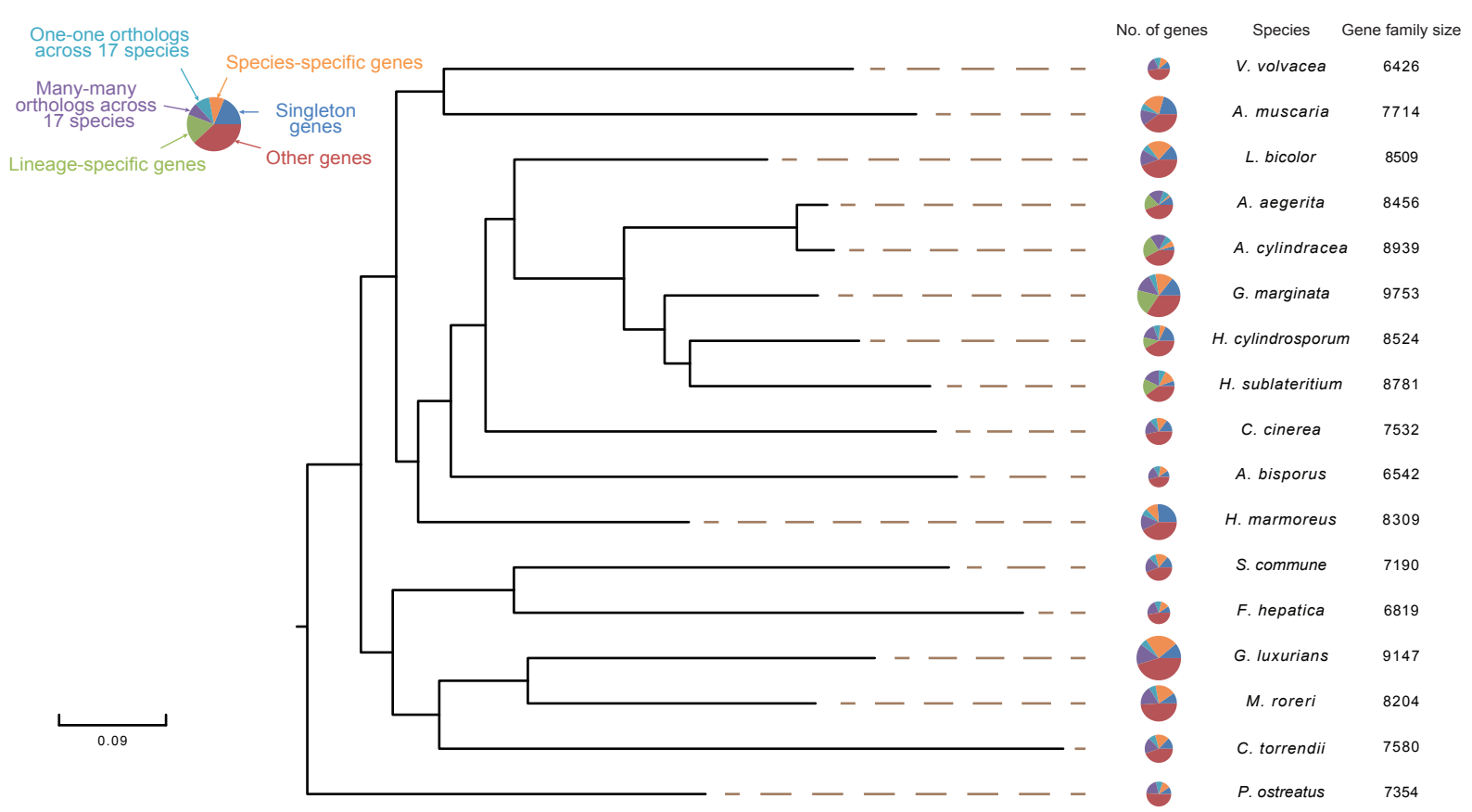

Supplement: Supplementary Figure S3 — Phylogenetic analysis of A. cylindracea and 16 Agaricales fungi based on single-copy orthologous genes. The phylogenetic tree was inferred based on RAxML from 315 orthologous proteins of the single-copy gene families. Serpula lacrymans serves as an outgroup. There is a close relationship between A. cylindracea and A. aegerita. [file mmc3.pdf]

*A. cylindracea*

*H. cylindrosporum*

*H. sublateritium*

*A. aegerita*

*G. marginata*

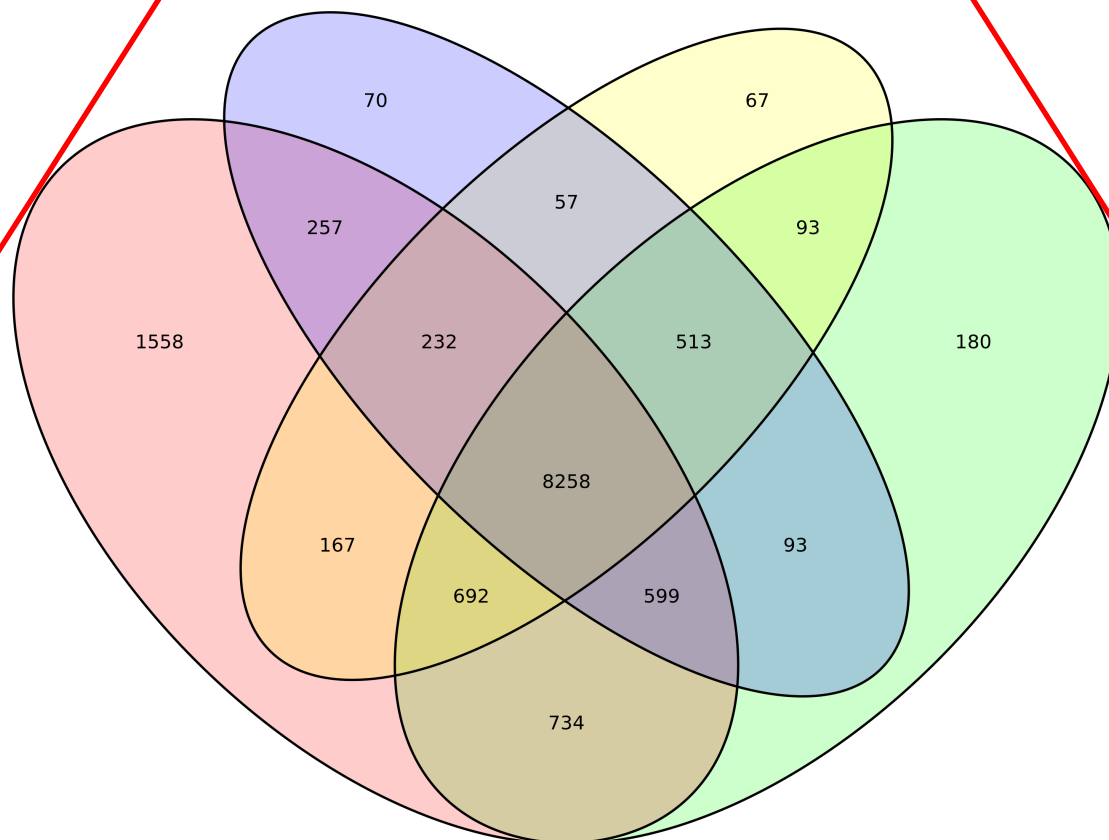

Supplement: Supplementary Figure S5 — Homologous genes shared among A. cylindracea, A. aegerita, G. marginata, H. cylindrosporum, and H. sublateritium The triangle represents the total genes of A. cylindracea. The ovals represent the shared homologous genes of A. aegerita (red), G. marginata (green), H. cylindrosporum (blue), and H. sublateritium (yellow) with A. cylindracea. [file mmc5.pdf]

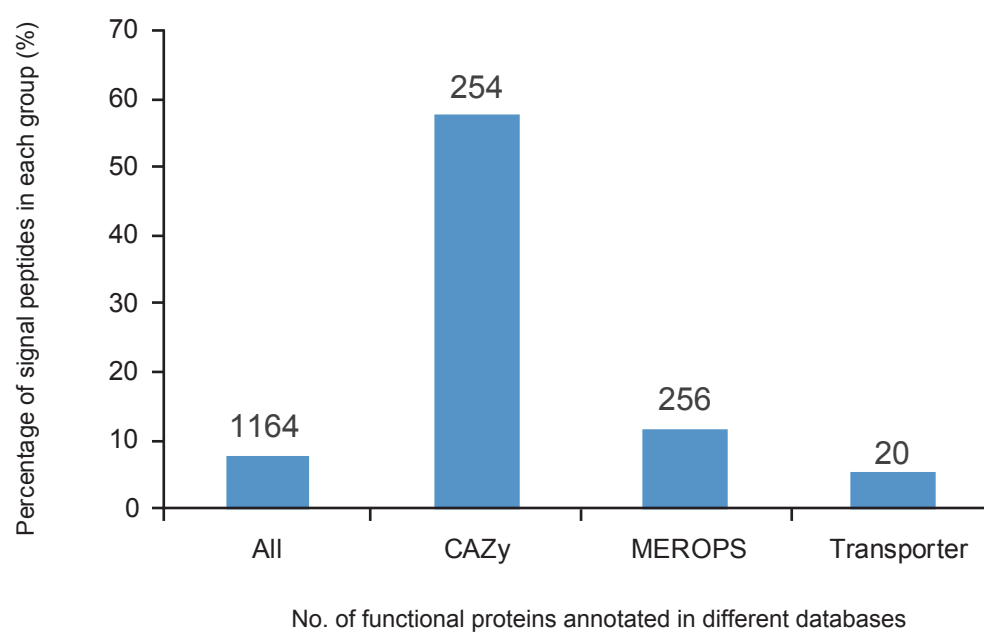

Supplement: Supplementary Figure S6 — Signal peptide ratio in A. cylindracea The vertical axis represents the percentage of proteins with signal peptides, and the horizontal axis represents functional proteins annotated by different databases: “All” represents all the predicted proteins in A. cylindracea; “CAZy” represents the carbohydrate hydrolytic enzymes in A. cylindracea based on the CAZy database; “MEROPS” represents the proteases in A. cylindracea based on the MEROPS database; “Transporter” represents the transporter-related proteins according to gene annotation in NCBI-NR database. The numbers above the columns represent the number of proteins with signal peptides in various databases. [file mmc6.pdf]
