# Supplementary material for: Genome Assembly and Pathway Analysis of Edible Mushroom Agrocybe cylindracea
Source: Genomics Proteomics Bioinformatics. 2020 Jun 17;18(3):341–51. doi: 10.1016/j.gpb.2018.10.009 (PMC7801210; doi:10.1016/j.gpb.2018.10.009)
Supplement: Supplementary Figure S4 — GO enrichment of A. cylindracea-specific genes compared with A. aegerita, G. marginata, H. cylindrosporum, and H. sublateritium Each circle represents a family of GO terms, and the circle size reflects the number of enrichment genes. The arrows point from parental GO terms to the filial GO terms. The darker the color is, the more significant the enrichment is. A. GO enrichment analysis of A. cylindracea-specific genes compared with A. aegerita in the BP category. B. GO enrichment analysis of A. cylindracea-specific genes compared with A. aegerita in MF category. C. GO enrichment analysis of A. cylindracea-specific genes compared with the other four fungi in the BP category. D. GO enrichment analysis of A. cylindracea-specific genes compared with the other four fungi in the MF category. BP, biological process; MF, molecular function. [file mmc4.pdf]

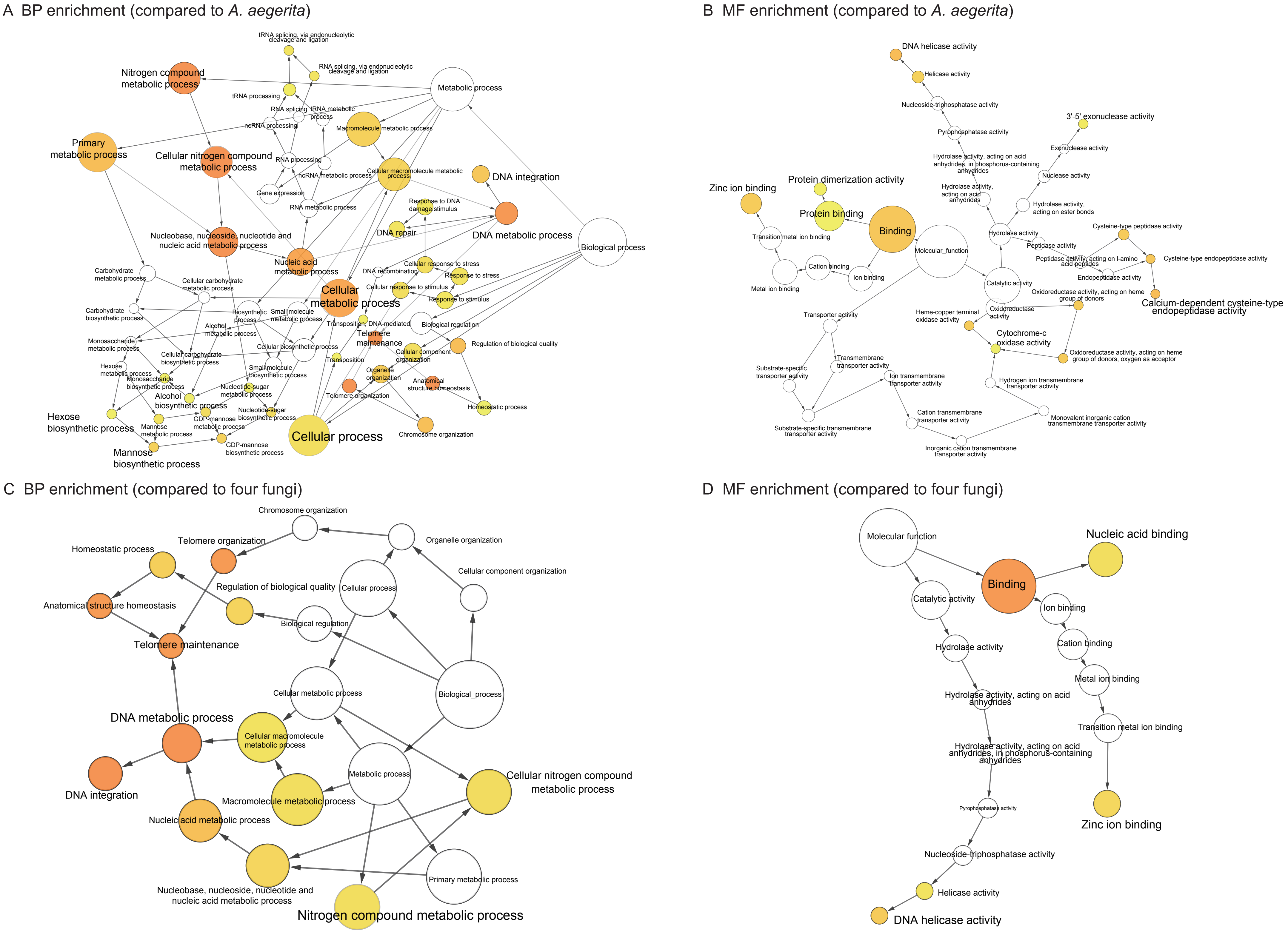
A BP enrichment (compared to A. aegerita)
tRNA splicing, via endonucleolytic cleavage and ligation
RNA splicing, via endonucleolytic cleavage and ligation
Nitrogen compound metabolic process
Metabolic process
tRNA processing
RNA splicing
tRNA metabolic process
ncRNA processing
Macromolecule metabolic process
Primary metabolic process
Cellular nitrogen compound metabolic process
RNA processing
Cellular macromolecule metabolic process
DNA integration
ncRNA metabolic process
Gene expression
RNA metabolic process
Response to DNA damage stimulus
DNA repair
DNA metabolic process
Biological process
Nucleobase, nucleoside, nucleotide and nucleic acid metabolic process
Nucleic acid metabolic process
Cellular response to stress
Carbohydrate metabolic process
DNA recombination
Response to stress
Cellular carbohydrate metabolic process
Cellular response to stimulus
Cellular metabolic process
Response to stimulus
Carbohydrate biosynthetic process
Biosynthetic process
Small molecule metabolic process
Transposition, DNA-mediated
Biological regulation
Alcohol metabolic process
Telomere maintenance
Monosaccharide metabolic process
Cellular biosynthetic process
Regulation of biological quality
Cellular carbohydrate biosynthetic process
Transposition
Cellular component organization
Hexose metabolic process
Small molecule biosynthetic process
Organelle organization
Monosaccharide biosynthetic process
Nucleotide-sugar metabolic process
Anatomical structure homeostasis
Alcohol biosynthetic process
Telomere organization
Homeostatic process
Hexose biosynthetic process
Nucleotide-sugar biosynthetic process
Mannose metabolic process
GDP-mannose metabolic process
Cellular process
Chromosome organization
GDP-mannose biosynthetic process
Mannose biosynthetic process
B MF enrichment (compared to A. aegerita)
DNA helicase activity
Helicase activity
Nucleoside-triphosphatase activity
3'-5' exonuclease activity
Pyrophosphatase activity
Exonuclease activity
Hydrolase activity, acting on acid anhydrides, in phosphorus-containing anhydrides
Protein dimerization activity
Nuclease activity
Zinc ion binding
Hydrolase activity, acting on acid anhydrides
Protein binding
Hydrolase activity, acting on ester bonds
Binding
Cysteine-type peptidase activity
Transition metal ion binding
Hydrolase activity
Peptidase activity
Molecular_function
Peptidase activity, acting on l-amino acid peptides
Cysteine-type endopeptidase activity
Cation binding
Endopeptidase activity
Ion binding
Catalytic activity
Metal ion binding
Oxidoreductase activity, acting on heme group of donors
Calcium-dependent cysteine-type endopeptidase activity
Oxidoreductase activity
Heme-copper terminal oxidase activity
Transporter activity
Cytochrome-c oxidase activity
Oxidoreductase activity, acting on heme group of donors, oxygen as acceptor
Substrate-specific transporter activity
Transmembrane transporter activity
Ion transmembrane transporter activity
Hydrogen ion transmembrane transporter activity
Cation transmembrane transporter activity
Monovalent inorganic cation transmembrane transporter activity
Substrate-specific transmembrane transporter activity
Inorganic cation transmembrane transporter activity
C BP enrichment (compared to four fungi)
Chromosome organization
Organelle organization
Telomere organization
Homeostatic process
Cellular component organization
Regulation of biological quality
Cellular process
Anatomical structure homeostasis
Biological regulation
Telomere maintenance
Cellular metabolic process
Biological_process
DNA metabolic process
Cellular macromolecule metabolic process
Metabolic process
Cellular nitrogen compound metabolic process
DNA integration
Macromolecule metabolic process
Nucleic acid metabolic process
Primary metabolic process
Nucleobase, nucleoside, nucleotide and nucleic acid metabolic process
Nitrogen compound metabolic process
D MF enrichment (compared to four fungi)
Molecular function
Nucleic acid binding
Binding
Catalytic activity
Ion binding
Hydrolase activity
Cation binding
Metal ion binding
Hydrolase activity, acting on acid anhydrides
Transition metal ion binding
Hydrolase activity, acting on acid anhydrides, in phosphorus-containing anhydrides
Pyrophosphatase activity
Zinc ion binding
Nucleoside-triphosphatase activity
Helicase activity
DNA helicase activity
