# Supplementary material for: Genome Assembly and Pathway Analysis of Edible Mushroom Agrocybe cylindracea
Source: Genomics Proteomics Bioinformatics. 2020 Jun 17;18(3):341–51. doi: 10.1016/j.gpb.2018.10.009 (PMC7801210; doi:10.1016/j.gpb.2018.10.009)
Supplement: Supplementary Figure S7 — Consistency between biological replicates of the A. cylindracea transcriptomic data. Both pilei (A, B, and C) and stipes (D, E, and F) of A. cylindracea fruiting bodies have three biological replicates. Graphics reveal a very high consistency between biological replicates: pilei_1, pilei_2, and pilei_3 are the biological replicates of the pilei, and stipes_1, stipes_2, and stipes_3 represent the biological replicates of the stipes. FPKM represents the gene expression level. The most highly expressed gene in pilei encodes aegerolysin (Aa-Pri1), while the most highly expressed gene in stipes encodes calmodulin. [file mmc7.pdf]

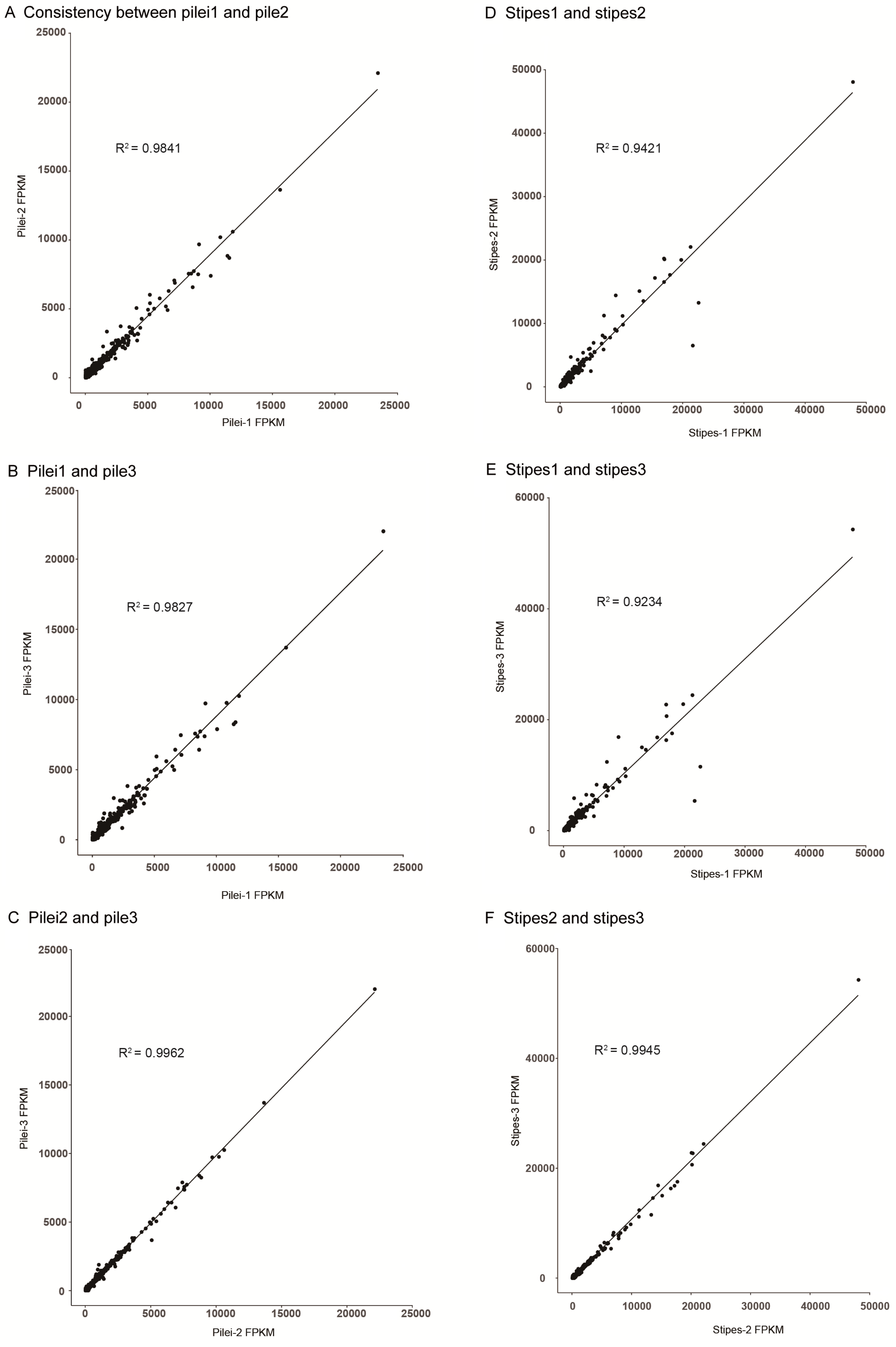
A Consistency between pilei1 and pile2
R2 = 0.9841
Pilei-2 FPKM
Pilei-1 FPKM
0
5000
10000
15000
20000
25000
D Stipes1 and stipes2
R2 = 0.9421
Stipes-2 FPKM
Stipes-1 FPKM
0
10000
20000
30000
40000
50000
B Pilei1 and pile3
R2 = 0.9827
Pilei-3 FPKM
Pilei-1 FPKM
E Stipes1 and stipes3
R2 = 0.9234
Stipes-3 FPKM
Stipes-1 FPKM
60000
C Pilei2 and pile3
R2 = 0.9962
Pilei-3 FPKM
Pilei-2 FPKM
F Stipes2 and stipes3
R2 = 0.9945
Stipes-3 FPKM
Stipes-2 FPKM
